# The genetic basis of major depressive disorder

**DOI:** 10.1038/s41380-023-01957-9

**Published:** 2023-01-26

**Authors:** Jonathan Flint

**Affiliations:** Department of Psychiatry and Biobehavioral Sciences, Billy and Audrey Wilder Endowed Chair in Psychiatry and Neuroscience, Center for Neurobehavioral Genetics, 695 Charles E. Young Drive South, 3357B Gonda, Box 951761, Los Angeles, CA 90095-1761 USA

**Keywords:** Neuroscience, Genetics

## Abstract

The genetic dissection of major depressive disorder (MDD) ranks as one of the success stories of psychiatric genetics, with genome-wide association studies (GWAS) identifying 178 genetic risk loci and proposing more than 200 candidate genes. However, the GWAS results derive from the analysis of cohorts in which most cases are diagnosed by minimal phenotyping, a method that has low specificity. I review data indicating that there is a large genetic component unique to MDD that remains inaccessible to minimal phenotyping strategies and that the majority of genetic risk loci identified with minimal phenotyping approaches are unlikely to be MDD risk loci. I show that inventive uses of biobank data, novel imputation methods, combined with more interviewer diagnosed cases, can identify loci that contribute to the episodic severe shifts of mood, and neurovegetative and cognitive changes that are central to MDD. Furthermore, new theories about the nature and causes of MDD, drawing upon advances in neuroscience and psychology, can provide handles on how best to interpret and exploit genetic mapping results.

## Introduction

In this review I consider what is known about the genetic basis of major depressive disorder (MDD), focusing on molecular genetic studies from 2015 onwards (predominantly genome-wide association studies (GWAS)). Previous reviews summarize earlier work [1, 2] and cover the unproductive, and sometimes contentious, history of candidate gene studies, including conflicting claims over the presence of gene-by-environment interactions [3]. The entire field of psychiatric genetics has moved beyond the candidate-gene and candidate-gene-by-environment approach, recognizing that these previous approaches relied on the existence of common genetic variants with large effects, a hypothesis that has now been abandoned. In its place stand the results from a series of GWAS, of which those addressing the genetic basis of MDD are summarized in Table 1.

**Table 1:** A summary of GWAS analysis of MDD.

Reference	Year	Population	Diagnostic definition	Cases	Controls	Loci
[144]	2009	Netherlands	DSM IV MDD	1783	1802	0
[145]	2010	Germany	DSM IV MDD	409	541	0
[146]	2010	Munich	DSM IV MDD (recurrent)	1359	1782	0
[147]	2010	UK	DSM IV MDD (recurrent)	1636	1594	0
[148]	2011	US	DSM IV MDD	3957	3428	0
[149]	2011	Germany	DSM IV MDD (recurrent early onset)	1020	1636	0
[150]	2011	Germany	DSM IV MDD	353	366	0
[151]	2012	European	DSM IV MDD	5763	6901	0
[152]	2013	PGC	DSM MDD	9240	9519	0
[35]	2015	China	DSM IV MDD (recurrent) women only	5303	5337	2
[7]	2016	23&Me	Broad depression (single item question)	75,607	231,747	17
[32]	2018	PGC + 23&Me	Mixed	135,458	344,901	44
[153]	2018	UK Biobank	Broad depression (single item question)	113,769	208,811	14
[153] [8]	2018 2019	UK Biobank PGC, 23&Me, UK Biobank	probable MDD	30,603	143,916	2
			ICD code for MDD	8276	209,308	1
			Broad depression (single item question)	246,363	561,190	102
[5]	2021	US Veterans (Europeans and African Americans)	ICD code for MDD	366,434	847,433	178
[30]	2021	China	Mixed	15,771	178,777	1
[20]	2021	Australia	DSM V (self report online)	13,318	12,684	1

In the population column, studies are classified by the source of their sample. PGC refers to the Psychiatric Genomics Consortium and 23&Me to the consumer genetics company. The column headed loci gives the number of genome-wide significant loci that each GWAS reports.

**Fig. 1:**
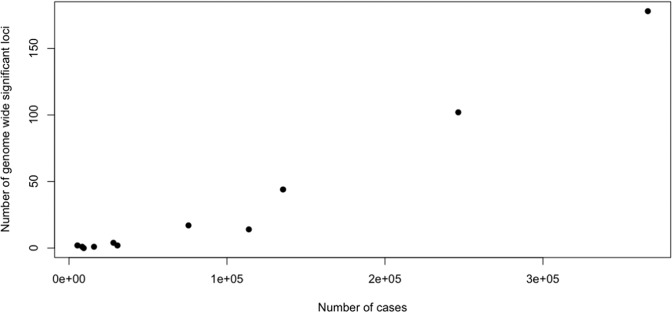
The relationship between sample size and the number of genome-wide significant loci. The relationship between the number of cases of MDD (plotted on the horizontal axis) to the number of genome-wide significant loci discovered (plotted on the vertical axis). Each dot represents the findings from a GWAS study.

Table 1 includes information on the number of cases and controls used by each GWAS, from which it can be see that success, defined in terms of number of loci identified, came with increases in sample size. There is an approximately linear relationship between the number of cases and the number of loci identified (illustrated in Fig. 1; for a discussion of the relationship between sample size and loci detected see [3, 4]). In short, the larger sample sizes have delivered more genome wide significant risk loci.

The sample sizes are large, even by current standards: the most recent GWAS (from 2021) analyzed data from 1.2 million participants to identify 178 genetic risk loci and 223 independently significant single-nucleotide polymorphisms (SNPs) [5]. Recruiting cohorts on this scale was made possible by using simple and cheap methods to identify cases, methods described in more detail below and which I shall refer to as minimal phenotyping. Realizing that large samples were necessary to obtain robust statistical significance for genetic association, genetic researchers adopted minimal phenotyping strategies on the assumption that even if the phenotype were measured poorly, association would still be detectable for some of the loci contributing to the genetic risk of MDD. As hoped, hundreds of genome-wide significant loci have been found, but the loss of specificity consequent upon the use of minimal phenotyping had a penalty: a large proportion of the signal identified isn’t attributable to MDD, making it hard to use GWAS findings to understand the biology of MDD.

I will discuss below why the current state of MDD genetics is problematic by reviewing the nature of the phenotype that has been mapped, the nature of the loci that have been identified, how minimal phenotyping definitions relate to the gold standard definition of MDD (structured interview to elicit DSM criteria by a clinically experienced interviewer) as well as to other psychiatric conditions, and finally turn to consider ways forward to develop robust genetic analyses of the world’s leading cause of disability [6].

## Most cases in GWAS of MDD have not been shown to meet criteria for MDD

As shown in Table 1, before the 2016 GWAS report from the consumer genetics company 23&Me [7] almost all cases were required to meet DSM criteria (though not all were assessed by clinical interview, and different assessment schedules were applied, a complication that I return to later). Studies after 2016 include many cases recruited by methods that do not assess DSM or ICD criteria for MDD. For instance, out of 246,363 cases in one large GWAS from 2019 [8], 82% were recruited by self-report of depression:127,552 individuals from the UK Biobank who replied yes to the question ‘Have you ever seen a general practitioner for nerves, anxiety, tension or depression?’ or ‘Have you ever seen a psychiatrist for nerves, anxiety, tension or depression?’ and 75,607 cases all diagnosed by answering a single item: “Have you ever been diagnosed with clinical depression?” (answers: “Yes”, “No”, “I’m not sure”). The same study used a replication sample of 414,055 cases, all of which were recruited in this way [8]. Similarly, the most recent large-scale GWAS [5] recruited 340,591 cases of which 89% were defined as cases through a minimal phenotyping strategy that did not interrogate whether subjects met either DSM or ICD criteria.

How many of the cases recruited from minimal phenotyping do meet MDD criteria? We can estimate this from the literature on single item screening tests for MDD: from this we learn that more than half of the cases identified from a single item result are false positives [9]. Short, two-to-three item questionnaires, perform a little better, but only four out of 10 participants who score positive are depressed, and six out of 10 are false positives. It’s reasonable to assume that more than half of the cases in GWAS for MDD, recruited by these simple one or two item assessments, don’t have MDD.

GWAS cases are also recruited by asking about the presence of depressive symptoms, and by examination of electronic health records or deployment of online questionnaires seeking to detect whether a subject meets DMS or ICD criteria. These methods also perform poorly in detecting cases of MDD. For example, case definition in the Million Veteran Program in part used the two-item PHQ scale that asks about the presence of depressive symptoms in the past 2 weeks [5]. This, and similar assessments, assumes that depressive symptoms and MDD overlap. Do they? Detecting depressive symptoms, diagnosing MDD, and making a diagnosis of lifetime MDD are not the same things. A diagnosis of MDD requires 2 weeks of clinically significant dysphoria or anhedonia, along with a total of five symptoms. Lifetime MDD is diagnosed by asking about the occurrence of MDD at any point in a subject’s life. One way to see the difference between MDD and depressive symptoms is from their respective prevalences. While up to 20% of community-ascertained adults admit to experiencing depressive symptoms in the previous 6 months [10], the prevalence of MDD that satisfies DSM criteria (diagnosed from structured interviews) is between 2 and 4% [11]. The 12-month prevalence of MD in the US, similarly diagnosed, is 6.6% [12], and lifetime prevalence in the US is estimated to be 16.6% for DSM-IV [13]. The differences between the high prevalence of depressive symptoms from screening scales and the lower prevalence of depressive disorders indicates that there are many people who do not meet diagnostic criteria for MDD, but do have some form of subsyndromal disorder. The relationship of this condition (or conditions) to MDD is poorly understood, though we do know that subsyndromal depression is a strong predictor of the subsequent onset of MDD [14]. The inclusion of these people in GWAS of MDD contaminates case definition, but by how much we do not currently know. The consequences, though, *are* known: reduction in the specificity of the genetic signal, as discussed later.

Electronic health records are an alternative source of cases. Rigorous evaluation of their accuracy in detecting MDD cases is lacking. We know that ICD codes (the usual features extracted) have low specificity in the US, largely because clinicians may bill an ICD code for a diagnosis on clinical suspicion rather than for confirmation of disease [15]. Unsurprisingly, attempts to identify patients with MDD from electronic health records conclude that the data inadequately capture diagnoses [16]. We don’t have side-by-side comparison of EHR diagnoses and diagnoses obtained from a structured interview carried out by a clinically experienced interviewer (the gold standard), but using a primary care physician’s diagnosis as a comparator, ICD codes were found to have 77% sensitivity and 76% specificity [17], also supported by analysis of ICD codes from 5487 individuals [18].

Do more detailed self-assessments perform any better, as some claim [19, 20]? The UK Biobank [21], the Australian Genetics of Depression Study [20, 22] and the UK based Genetic Links to Anxiety and Depression Study [23] have all used a version of the CIDI-SF [24]. MDD assessed by the online CIDI-SF has higher heritability and captures more of the genetic signal that is specific to depression than briefer assessments [25], but we lack data comparing MDD diagnosed by gold-standard structured interview with the CIDI-SF (one conference report gives a validation of 81.8% for diagnosing recurrent MDD [26]). There is one report in the literature comparing MDD diagnosed by interviews and by a detailed self-assessment: the 20 item Centre for Epidemiological Studies Depression Scale (CES-D) [27]. About a third of cases with MDD were missed, and one third of those exceeding the CES-D threshold were diagnosed at interview with MDD [28]. In summary, longer self-assessments perform better than shorter ones, but we lack rigorous evaluation of their performance in large scale genetic studies. I turn to consider whether the low specificity matters, and argue that it does.

## The majority of the genetic risk loci identified with minimal phenotyping approaches are unlikely to be MDD risk loci

It’s sometimes claimed that MDD cases identified by minimal phenotyping are just less severe forms of MDD, and thus share the same genetic loci [1]. That would be equivalent to lowering the threshold for disease liability in the population above which “cases” for MDD are defined. Under the liability-threshold model [29] lowering the threshold would not reduce heritability assessed by single-nucleotide polymorphisms (h^2^_SNP_), yet h^2^_SNP_ estimated for the minimal phenotyping definitions of MDD is less than that for the well-defined: three studies have estimated the heritability of severe recurrent depression to be about 25%, compared to <10% for symptom-based depression [5, 25, 30].

It can also be argued that GWAS of a poorly defined phenotype might not matter if it could be shown that the loci identified index a remitting and often relapsing history of episodes of disturbances of sleep and appetite, suicidality, guilty ruminations, anhedonia and low mood, in short, the features that clinicians would want to target for treatment. Minimal phenotyping approaches perform poorly in finding such loci. We know this from two analyses, using different strategies in different samples, that addressed the question of the specificity of genetic action in studies of MDD.

The first analysis applied a minimal phenotyping definition of MDD in 10,148 twin samples from three independent studies, and then estimated the fraction of genetic effects specific to lifetime MDD (as diagnosed by structured interviews by carefully trained mental health professionals [31]) that is captured by a less well-characterized case definition. The minimal phenotyping definition was more detailed than the single item assessments mentioned above, as it included self-administered questionnaires of current depressive symptoms and the personality trait neuroticism, both of which measure negative affect (central to the concept of MDD). Nevertheless, even this broad phenotype would miss around 65% of the risk loci for MD, including those specific to the syndrome [31]. Single item assessments, containing less information than the broad definition used here, likely index even less of the MDD-specific genetic risk.

A similar conclusion came from a second study which used SNP-based analysis of heritability (h^2^_SNP_), comparing single-item, self-reported treatment seeking for depression with “Lifetime MDD”, defined using answers to a longer questionnaire (both the CIDI-SF and PHQ9) [25] that contained nearly all of the individual DSM criteria. Again, the majority of the heritability of the more strictly defined MDD is not shared with the lightly phenotyped measure [25]. The loss of signal unique to MDD is again likely underestimated, because “Lifetime MDD” did not come from a structured interview administered by a clinically trained interviewer, the gold-standard for MDD diagnosis.

**Fig. 2:**
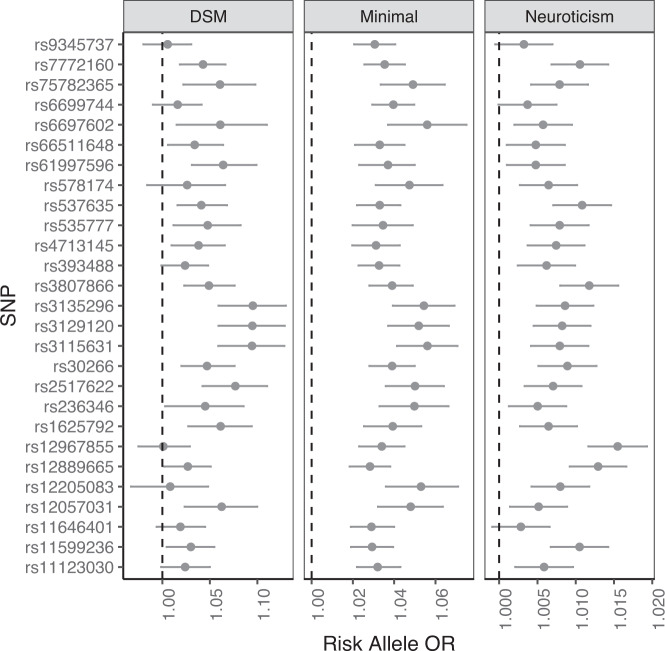
GWAS hits from minimal phenotyping definition of MDD in UKBiobank are not specific to MDD. The figure shows 27 loci, listed on the vertical axis, that are significantly associated with a minimal-phenotyping definition of MDD in UKBiobank (GPpsy). The odds ratios (OR) are shown on the horizontal axis for the minimal phenotype, for a DSM-diagnosis of MDD and for the personality trait, neuroticism. The latter is a quantitative phenotype, so to allow comparison with the binary traits, the effect size estimates from the regression (beta values) have been converted into odds ratios. Data are from [25].

The lack of specificity can be seen by comparing the loci mapped by minimal phenotyping with those mapped by other traits. Once we have identified risk loci from a minimal phenotyping definition of MDD, we can ask how many of them also increase the risk for more strictly defined MDD. The answer is shown in Fig. 2. In the middle are the effects (plotted as odds ratios) for genome-wide significant loci found from a minimal phenotype definition (“GPpsy”) mapped in UK Biobank (data from [25]). On the left of the figure are the effects of the same loci on a “Lifetime MDD” definition. Consistent with the expectation that the same loci contribute to both traits, the effects at each locus are in the same direction and most are significant.

However, Fig. 2 also shows that the loci identified by mapping a minimal phenotyping definition MDD contribute to the personality trait neuroticism, plotted on the right of the diagram. In other words, the strategy has identified non-specific loci. Mapping minimal phenotyping MDD has identified loci shared with neuroticism, and not those that are specific to MDD. We could go ahead and characterize these loci, to identify the biology that they index, but if we did so and use the results to help us design better treatments for MDD, then we can expect those treatments also to affect the personality trait of neuroticism.

The results described above don’t mean that all loci mapped for the minimal phenotyping definition are non-specific. There will be some that also index the features of MDD we are interested in. But how can they be identified? In the absence of a well powered GWAS of MDD (diagnosed by interview) we can’t distinguish specific from non-specific genetic effects.

## Genetic correlations between MDD definitions do not demonstrate that the definitions have the same biological basis

An appealing way to validate the use of minimal phenotyping definitions is to use SNP-based methods to demonstrate pleiotropy (i.e., that two traits have the same genetic basis). We could, for example, measure the genetic correlation (rG_SNP_) between definitions of MDD and determine how much of the genetic effects are common to the different definitions. This strategy was used to compare data from seven cohorts that made up a GWAS of 135,458 MDD cases and 344,901 controls [32], and the genetic correlations were interpreted to support “the comparability of the seven cohorts (Supplementary Table 3), as the weighted mean rG_SNP_ was 0.76 (s.e. = 0.03)”. [33]

There are three problems with this conclusion. First, the estimates are similar in magnitude to those between MDD and other phenotypes, particularly with other internalizing constructs. Table 2 shows the correlations between the seven cohorts, taken from [32]. Supplemental Table 13 of the same paper reports rG_SNP_ with neuroticism of 0.7 (se 0.03) and 0.67 (se 0.04) with tiredness, both values larger in magnitude than some of the rG_SNP_ estimates between the MDD cohorts. If we use rG_SNP_ of the magnitude reported in Table 2 to justify the use of minimal phenotypes, we would have to admit measures of personality and tiredness to be equally valid measures of MDD. Second, the correlations depend on other features than just pleiotropy, making it hard to interpret a comparison of estimates between cohorts that have not been collected in the same way. Differences in ascertainment, in sex, and in age across cohorts alter the genetic architecture (this is discussed in the section on heterogeneity) [33]. Third, even if we accept the rG_SNP_ values as correct, then the value of 0.76 [32] means 43% (calculated as 1 - 0.76 squared) of MD risk variants are not shared among cohorts, clearly a problematic level. Overall, these considerations imply that using rG_SNP_ to make inferences about the biological relationships between MDD cohorts (and with other traits) may be confounded by other features, unrelated to the biology, which undermines the use of the measure to determine the biological similarity of the MDD definitions.

**Table 2:** Genetic correlations between seven cohorts used in GWAS (data taken from [32]).

Number of cases	Cohort	PGC29	deCODE	GenScot	GERA	iPSYCH	UK Biobank
16,823	PGC29						
1980	deCODE	0.97 (0.28)					
997	GenScot	0.83 (0.37)	0.76 (0.48)				
7162	GERA	0.59 (0.13)	1.11 (0.35)	0.35 (0.38)			
18,629	iPSYCH	0.59 (0.10)	0.85 (0.25)	0.58 (0.50)	0.84 (0.15)		
14,260	UK Biobank	0.96 (0.13)	1.17 (0.31)	1.10 (0.34)	0.97 (0.19)	0.69 (0.12)	
75,607	23&Me	0.67 (0.06)	0.80 (0.20)	0.40 (0.18)	0.94 (0.13)	0.78 (0.06)	0.80 (0.08)

The table gives the SNP based estimates of genetic correlation with their standard error in brackets.

Polygenic risk score (PRS) can also be used to test the relationship between two phenotypes. A PRS sums the genetic effects estimated in one cohort to predict disease status in another. We can ask whether a PRS from the minimal phenotyping definitions does well at predicting MDD in more well-defined cases. The answer to that question is that it depends on the sample size: as sample size increases, PRS accuracy increases (see Fig. 2a in [32]). However, the issue is not just accuracy. What we want to know is whether the PRS from minimal phenotyping performs as well, or better than that from better defined MDD in predicting MDD meeting DSM criteria in cases. The short answer is that it does not. Once samples of the same size are used, then a PRS from a better defined MDD out-performs the minimal phenotyping PRS [25]. Putting this observation together with the analysis of non-specific effects above, then we can conclude that increasing the sample size will increase the ability to predict the mostly non-specific genetic components of MDD, although a modest proportion of genetic risk specific to MDD will also be well predicted.

## What has been mapped?

Almost all GWAS have mapped a vulnerability to low mood or negative affect, a trait which is best termed dysphoria, to distinguish it from MDD. The genetic basis of dysphoria is in part shared with MDD, but (and this is the critical argument) there is a large genetic component unique to MDD, inaccessible to minimal phenotyping strategies. This includes the cyclic shifts of mood episodes and neurovegetative and cognitive changes central to MDD for which we lack adequate treatment.

If there are genetic effects unique to MDD, distinguishing it from dysphoria, is there any evidence that the genes involved, the biological pathways, are different? It’s too early to draw any definitive conclusions from the available data, not just because it is a hard task to conclusively find genes [34] but because we have so few results from GWAS for rigorously defined MDD. One study has identified and replicated two genome-wide significant loci in a sample of women with recurrent MDD, meeting diagnostic criteria as determined at structured interview [35]. While this is not enough to draw any conclusions, additional loci emerged from analysis of gene by environment interaction [36] and from analysis of rare variants identified from low-coverage sequence data [37]. Candidate genes identified from these separate analyses are enriched in mitochondrial function, supporting observations of increased amounts of mitochondrial DNA in cases [38, 39].

By contrast, genes implicated by GWAS of dysphoria are enriched in neurodevelopmental functions. The two most recent GWAS [5, 20] derive candidate gene lists based on the proximity of risk loci to genes (using a computational approach [40]), and on association with variation in transcript abundance [41]. The two candidate gene lists share 64 entries. Of the 64 genes present in both lists, twelve (almost 1 in five) contain zinc finger domains (ZNFs, ZSCANs and ZKSCANs). Zinc fingers recognize specific DNA sequences, with consequences that depend on other motifs in the protein, but typically involving the regulation of gene transcription, often in development. Although common, the 3% of genes in the human genome that contain them is far less than the almost 20% of genes in the dysphoria gene lists. Furthermore, the presence of three protocadherins (PCDHA1, PCDHA1 and PCDHA3) together with PAX6, supports the implication from the zinc finger genes of the role of developmental mechanisms, in particular involving neurons (a target of protocadherin function). Perhaps unsurprisingly the most significant functional category among the genes is “Nervous system development” [8].

## More information is needed than just a DSM diagnosis

Given the difficulties of obtaining sufficient cases that meet diagnostic criteria for MDD, it might seem churlish to complain that isn’t enough for genetic studies. If GWAS studies recruited cases based on DSM criteria, a more standardized and reliable phenotype than self-assessments will be mapped, but one which may still have little or no relationship to any underlying biological entity. Indeed, DSM-5 is explicitly atheoretical, making no claim that the depression it describes reflects known neurobiological, or indeed any other, psychological process.

The dangers of concentrating solely on meeting DSM criteria have been recognized for some time: Hyman noted in 2007 “The problematic effects of diagnostic reification were revealed repeatedly in genetic studies, imaging studies, clinical trials, and types of studies where the rigid, operationalized criteria of the DSM-IV defined the goals of the investigation despite the fact that they appeared to be poor mirrors of nature” [42]. After a detailed review of the diagnostic features of MDD, Kendler points out that “meeting the DSM criteria for major depression is not the same thing as having major depression” [43], and that we are in danger of becoming “stymied by an excessive respect for our own creation” [43].

Another way to express this problem is as follows. As explained above, between 60 and 75% of the genetic risk for interview-based lifetime MDD is unique [31]. If we just map cases with DSM-diagnosed depression, obtained by gold-standard methods, we won’t be able to decide which of the loci we find are unique to MDD (in the sense described above). MDD arises more from environmental than from genetic roots, with a complex and poorly understood set of interactions between the two; the disorder is highly comorbid with other psychiatric disorders and with chronic disease; differences in personality, sex and age all contribute differentially to the risk of developing the illness [44, 45]. That complexity has to be incorporated into genetic analysis if we are to adequately interpret GWAS results.

## MDD is likely heterogeneous

A complication for the genetic analysis of MDD, and one that strongly indicates the need for us to collect more information than the diagnosis, is that multiple lines of evidence indicate the disorder is heterogeneous. Clinical features [46–50], comorbidities (~75% of patients with depression will meet criteria for at least one additional psychiatric disorder [51]), co-occurrence of diagnostic biomarkers [52–54], clustering subjects according to shared signatures of brain function [55–57] and treatment response [58–60], all point to this conclusion, although there is no agreement on, or conclusive demonstrations of, what the subtypes are [61, 62]. There is a large literature on this question, including a recent comprehensive review of genetic heterogeneity [63]. I will focus here on issues relevant to interpreting GWAS findings.

First the genetic contributions to MDD subtypes are likely to differ. There is considerable empirical support for such a view. Most [64–66], but not all studies [67] find evidence for a higher heritability for major depression in women than in men, and also report that the genetic effects are not completely shared between the sexes. The largest study of the correlation in genetic effects, using 1.7 million pairs of monozygotic and dizygotic twins and full and half siblings [65], estimated the correlation to be 0.89 (95% CI = 0.87, 0.91), consistent with two earlier, smaller twin studies [68, 69]. There is also evidence that cases ascertained through hospitals have a higher heritability than community acquired cases [70], that there is higher heritability for recurrent MDD compared to single episode illness, and for early onset compared to later onset [71–77]. Conversely, stratifying cases by clinical features, patterns of comorbidity, recurrence and age of onset, identifies differences in SNP-heritability, as was found in the UK Biobank, where genetic correlations between clinically defined subtypes ranged from 0.55 to 0.86 [78].

The presence of genetic heterogeneity has important consequences for interpreting GWAS studies. It means groups ascertained under different protocols will not share the same genetic risk loci, as demonstrated from the genetic correlations between 29 cohorts from the Psychiatric Genomics Consortium (PGC): rG_SNP_ estimates varied from 0.52 to 1 (Supplementary Table 2 [32]). Genetic analysis carried out in ignorance of the presence of subtypes, as will happen with studies that use minimal phenotyping, enriches non-specific signals. Large sample sizes will eventually overcome sample heterogeneity [32], but at the cost of losing signal that is specific to the disease.

Ignoring subtypes can also introduce discrepancies between studies. As an example, a meta-analysis of 6561 cases found a significant inverse association between MDD and an obesity risk variant (in an intron of the FTO gene [79]; odds ratio = 0.92 (0.89, 0.97), *P* = 3.0E−04) [80]. An independent sample failed to replicate the association, except by stratifying on clinical features, when the locus was found to *increase* the risk of atypical MDD (odds ratio = 1.42-fold, *P* = 1.84E−04) [81] (the ‘atypical’ subtype was differentiated mainly by the direction of change in appetite, weight and sleep [82]). The sample sizes are relatively small and the delineation of subtypes incomplete so we cannot draw firm conclusions from this finding, but it is an indication of what will happen if subtypes are not considered.

To what extent can genetic analysis validate subtypes? There are conflicting claims that it can detect subtypes [78], and also that it cannot [83, 84]. We can state with certainty that there is almost no evidence for the presence of experiments of nature, large mutations, that will cast light on the depression’s pathogenesis [2] (despite continuing hints that there are rare instances of single causes [85]) but there is much less certainty around what we can expect to be able to detect. The illustrative example here are attempts to stratify MDD by environmental risk. Given the size of the effect (more than half of the risk of developing depression is environmental [65]), stratifying by environmental risk should be a comparatively easy target. The fact that it is not, is itself instructive.

There’s an old distinction between ‘reactive’ depression, in which cases are caused by exposure to stressful life events, and ‘endogenous’ depression, in which no external cause can be found [86–88]. A putative precipitating event can be found for about half of MDD cases [89, 90], suggesting that additional factors are necessary for the adverse life event to result in a depressive episode. Are there genetic differences between those exposed and those not exposed to life adversity? One report has identified different risk loci in the two groups [36] but, in general, attempts to detect such heterogeneity have yielded contradictory results.

Almost all studies addressing this question resort to the use of a polygenic risk score (PRS), which sums the effects estimated in one cohort to predict disease status in another. The first such analysis, in a small (1645 MDD cases) well phenotyped sample from the Netherlands, found that PRS have limited impact in predicting MDD risk in individuals with little exposure to childhood trauma, but a large impact in individuals with high exposure to childhood trauma [91]. A second study (of 1605 MDD cases, again well phenotyped) showed the opposite: cases who experienced more severe childhood trauma had a lower PRS than other cases or controls [92]; a third study, using 3024 MDD cases from nine cohorts of the PGC, found no evidence of any significant interaction [93]. A recent analysis of UK Biobank patients used a genomic relationship matrix to capture genetic relationships rather than the PRS, found that genome-by-trauma interaction accounts for greater variance in male than female individuals [94] (though note that this result applies to the dysphoria phenotype, not MDD). Alternative approaches to investigating the impact of the environment are now being developed [95, 96] but robust replicated results are still lacking. The current literature is inconclusive, with no clearly replicable patterns emerging using current methods.

MDD heterogeneity is likely going to be very messy, due to environmental effects operating differently in different cohorts, with an altogether much richer and complex pattern of interactions, a degree of context dependency that we have not so far been able to measure. MDD may consist of many overlapping subtypes, that are only partly distinguishable based on clinical features, disease trajectory, risk factors, response to treatment and genetic risk factors. One instructive example where this possibility has been examined is inflammatory bowel disease in a model which supposes the existence of many environmental variables acting cumulatively over time on a backdrop of many genetic variants [97]. Testing whether MDD might similarly be best explained as a system-level perturbation of multiple, interacting factors, will require much larger, deeper datasets than are currently available.

## Genetic relationships between MDD and other traits

Every GWAS since 2016 has used the genotypes to examine the relationship between what is claimed to be MDD (what I have termed dysphoria) and other disorders. I’ve already illustrated the use of genetic correlations and polygenic scores to examining the relationship between different definitions of MDD; the same methods have been applied to examine the relationship between MDD and other psychiatric disorders, and indeed many other traits and diseases. Table 3 summarizes recent findings, providing data on SNP-based estimates of genetic correlation (rG_SNP_) and comparing them where possible to family based and twin-based estimates (rG-family and rG-twin) for four diseases and for the personality trait neuroticism (high neuroticism scores are robustly associated with an increased risk for MDD [98–100]).

**Table 3:** Genetic correlations between depression and four other psychiatric diseases (attention deficit hyperactivity disorder (ADHD), autism spectrum disorder (ASD), schizophrenia, bipolar disorder) and one personality trait (neuroticism).

Trait1	Trait2	rG_twin_ (s.e.)	rG_SNP_ (s.e.)	Ref
Major depressive disorder	Bipolar disorder	0.64 (0.06)		[154]
Dysphoria	Bipolar disorder		0.35 (0.03)	[101]
Dysphoria	Bipolar disorder		0.44 (0.02)	[155]
Dysphoria	Bipolar disorder		0.33 (0.03)	[8]
Dysphoria	Bipolar disorder		0.36 (0.03)	[156]
Dysphoria	Schizophrenia		0.32 (0.02)	[8]
Dysphoria	Schizophrenia		0.34 (0.03)	[101]
Dysphoria	Schizophrenia		0.34 (0.03)	[156]
Dysphoria	Autism		0.45 (0.04	[156]
Dysphoria	Autism		0.16 (0.06)	[101]
Dysphoria	ADHD		0.44 (0.03)	[156]
Dysphoria	ADHD		0.52 (0.06)	[101]
Dysphoria	Neuroticism		0.74 (0.04)	[101]
Major depressive disorder	Neuroticism	0.46 (no se)		[157]
Major depressive disorder	Neuroticism	0.43 (0.09)		[158]
Dysphoria	Neuroticism		0.21 (0.04)	[155]
Dysphoria	Neuroticism		0.70 (0.03)	[8]

The first column divides results for depression into MDD and for a related trait, dysphoria, as defined in the text. Genetic correlations are estimated from twin studies (rG_twin_) and from SNP-based methods rG_SNP_.

One interpretation of the rG_SNP_ findings in Table 3 is that they indicate the presence of pleiotropy, genetic loci that contribute to the risk of more than one disease, leading for example to the assertion that “genetically informed analyses may provide important ‘scaffolding’ to support restructuring of psychiatric nosology” [101]. The ease of generating rG_SNP_ results, which require only GWAS summary statistics, has led to an explosion of findings: 669 phenotypes were significantly genetically correlated with dysphoria in the most recent GWAS [5]. Before accepting this conclusion, we need to assess whether there are alternatives to pleiotropy as explanations for the rG_SNP._ findings.

A review of the interpretation of rG_SNP_ identified the following features that could bias estimates [102]: misclassification, assortative mating, population stratification, sample ascertainment (in particular ‘collider bias’ [103]) and inclusion of ‘super-normal controls’ [104]. All these probably affect the rG_SNP_ reported in Table 3, but I will focus here on three which likely make the largest contribution.

The first is mis-diagnosis. Cohorts are inevitably going to contain a proportion of misdiagnoses, as discussed in previous sections. Cross-contamination across two disorders inflates their apparent correlation, and cross-contamination of either with a third will alter the estimate, depending on the true genetic sharing between the third disorder and the two whose rG_SNP_ we are trying to measure. This has already been shown for alcohol consumption [105] but, for reasons due to the source of MDD cases for GWAS, we lack similar data for MDD.

A second factor is how subjects were recruited into a study (ascertainment). There’s a tendency to assume that just because we deal with genetic data, a classic epidemiological problem of ascertainment can be ignored: after all, genotypes are fixed at conception so their relationship with the phenotypes must be causal. Unfortunately, completely artifactual genetic correlations can arise if two unrelated traits bias recruitment. In the UK Biobank study, enrolment implicitly selected participants having higher educational status and lower prevalence of smoking than the general population, and this introduces a bias in the estimated rG_SNP_ between educational status and smoking [103]. The same biases will impact other rG_SNP_ estimates, but we don’t know by how much. The choice of diagnostic protocols plays a role here, for example inflating estimates between depression and neuroticism. In the UK Biobank (and presumably in other cohorts) the diagnosis of depression came from a phenotyping strategy that enriches for neuroticism [25]. When rG_SNP_ with neuroticism was estimated from a cohort with severe major depressive episodes (severe enough to warrant treatment with electroconvulsive therapy, often seen as treatment of last resort), then the rG_SNP_ estimate fell to 0.42, a value consistent with that obtained from twin data (Table 3) [106].

Finally, cross-trait assortative mating over even a few generations will inflate estimates of rG_SNP_. Assortative mating refers to people choosing their partners because of something they have in common, such as height. Cross-trait assortative mating operates across multiple traits: we choose our partners not only because they are, roughly, similar heights as us, but also because we have other features in common, our likes and dislikes, our educational attainment and so on. Assortative mating induces rG_SNP_ through gametic phase disequilibrium, resulting in positive correlations between independently inherited genetic risk factors [107, 108]. Under conditions of random mating the number of risk alleles on one chromosome does not predict the number of risk alleles on a different chromosome, but they can predict this in the presence of assortative mating: the test for assortative mating can be carried out by asking whether risk alleles on odd-numbered chromosomes predicts the number of risk alleles on the even-numbered chromosomes. This test has been applied to explore genetic correlations between MDD and other phenotypes [109].

Cross-trait assortative mating has a surprisingly large impact on estimates of genetic correlation between MDD and psychiatric disorders, and can on its own be sufficient to account for many of the findings. Adding in the possibility of mis-diagnosis, after five generations of assortative mating and with a 5% bidirectional misdiagnosis (a very conservative estimate) most of the genetic correlation between depression and schizophrenia can be attributed to assortative mating (Fig. S11 of [109]).

In short, to use rG_SNP_ findings to reveal shared genetic bases between MDD and other phenotypes we must consider misdiagnosis, ascertainment and cross-trait assortative mating, among other things. Currently no method does that. Consequently, at present we can’t use rG_SNP_ estimates to make claims about the extent to which MDD shares biological roots with other traits and diseases.

## Turning silver into gold

The poor quality of the phenotyping used in genetic studies of MDD goes largely unremarked. The problem is not just that the aggregate genetic signal in lightly phenotyped samples is substantially weaker, it’s that much of the signal is likely wrong [25]. Those unfamiliar with the difficulties of psychiatric diagnosis and of the literature on the reliability and interpretation of questionnaire-based assessments could be forgiven for believing claims that GWAS has revealed the position in the genome of hundreds of genetic risk variants to a disease that makes the single largest contribution to disability in the world [6]. They could also be forgiven for believing claims that the genetic data accumulated from MDD GWAS can be used to make inferences about genetic correlations between MDD and other phenotypes, and to derive genetic risk scores that can be used in out-of-sample prediction. I have argued here these claims are poorly supported by empirical data. In most cases, MDD case definition is so lax we really don’t know what has been mapped. For want of a better term, I’ve called it dysphoria, to distinguish it from MDD. The use of a poorly characterized phenotype, with low specificity for MDD, may mean that advances in MDD genetics turn out to be as poorly substantiated as the earlier claims for the role of candidate genes [110]. In this section I provide my opinion on how to ensure we take the discoveries we have, even if they are imperfect, and improve them, by turning silver into gold.

How can we recruit better cohorts for MDD genetics? One option is to deploy new technology to improve diagnosis. Computerized adaptive testing [111, 112] and digital technologies both provide novel opportunities [113]. A computerized adaptive diagnostic test fixes the number of items administered and allows measurement uncertainty to vary. It’s faster than questionnaires and one for MDD obtained sensitivity of 95% and specificity of 87%, using an average of 4 items per participant (with a maximum of just 6 items) [114]. Computerized adaptive diagnostics could improve specificity over many of the existing self-assessments, but their performance compared to structured-interview DSM diagnosis for genetic research is unknown. There has also been progress in using digital phenotyping to infer mood and depression from data collected from phones [115, 116] and assess current mood from voice and facial features [117, 118], but the relevant literature consists largely of reviews and of methodologies [116, 119], rather than transformative advances. There is some success, but nothing that would yet give us the equivalent of a diagnosis of lifetime MDD.

Another, simpler, option is to deploy better self-assessments, such as the CIDI-SF [20–23]. This approach will provide better diagnoses, but all self-assessments, however detailed, are to some extent flawed. Fried’s detailed review of MDD assessments argues that the processes involved when people self-score will influence depression measurement [120]. There is scant literature on this subject, but we know that self-assessments over-estimate the prevalence of depression [121–124], sometimes substantially (25% compared to 12% in a meta-analysis of individual participant data [124]).

In summary, relying on self-assessed MDD, even with multiple item questions, will likely always have relatively low specificity. As such, longer self-assessments cannot replace clinical interviews for MDD diagnosis in recruitment for GWAS studies. Simply increasing sample size using current online screening tools is not enough: we need also to increase the specificity of diagnosis. That raises three further issues: how big a sample of gold-standard cases do we need, who should we recruit, and what additional information should we collect?

We certainly won’t need to obtain hundreds of thousands of interview-based diagnoses. The very large numbers needed for genetic studies can be obtained by phenotypic imputation, a method in which we take a number of the deeply phenotyped subjects and use them to predict high quality MDD diagnoses, and other clinical features, in those for whom we have much less information. Phenotypic imputation has been successfully applied to several phenotypes [125] but its success depends on the quality of the observed data and the structure of missingness. We need a set of well phenotyped cases to seed imputation, but how to maximize imputation’s effectiveness remains an open question, so it’s not possible to provide robust estimates of the number of interview-based cases required. As an example of what is possible, imputation using data from a questionnaire-based measure of MDD from 67,164 UK biobank into 337,126 individuals with a single-item measure increased both the number of risk loci identified and out-of-sample prediction of MDD accuracy, while preserving better specificity to MDD than the single-item measure [126].

Who should we recruit? There are strong arguments to be made for the collection of samples of diverse ancestry, as laid out by Peterson and colleagues [127]. Given that almost 80% of participants in GWAS are of European descent [128], samples with greater ancestral diversity would help address health disparities in the use of genomic medicine [127], aid locus discovery and provide more generalizable polygenic risk scores. Sampling diverse populations is beginning, under an initiative from the US National Institute of Mental Health, so we can expect to have data soon that will address the current imbalance in ancestry.

There are also arguments to be made in favor of designing studies to collect specific groups of patients, focusing on one sex, on recurrent depression and hospital rather than community ascertainment. Analysis of between-cohort genetic heterogeneity using data from 29 independent component cohorts of the PGC-MDD demonstrated that cohort ascertainment (e.g., clinical versus community recruitment) in part explains heterogeneity in heritability estimates and genetic correlations [32]. Targeted recruitment would reduce heterogeneity and potentially increase relevant genetic signal, as shown in the CONVERGE cohort [35], where recruitment of women with recurrent MDD ascertained in hospitals (predicted to increase heritability and homogeneity), yielded a sample with heritability (h^2^_snp_) of ~25% [37], compared to about 9% reported by the (predominantly European) PGC-MDD group [32].

What additional information should be collected? I’ve stressed the need for more cases diagnosed by clinical interview, and then asserted that collecting cases that meet diagnostic criteria isn’t enough, since meeting DSM criteria is no guarantee of identifying a biological relevant entity [42, 43]. I’ve pointed out that genetic risk loci for DSM-diagnosed MDD consist of a mix of loci specific for the condition and those that are not. We still need to distinguish loci that are specific from those that are non-specific, and to do that we need more data than case status alone. What information should we collect, so as to avoid the problem of reification [129], and allow us to identify loci that are specific to MDD?

The Australian Genetics of Depression Study provides one example of a set of additional phenotypes that could be acquired [20, 22]. These include comorbid disease (other psychiatric conditions, particularly anxiety disorders, [12] as well as medical disease [130]), environmental stressors, personality, family history, demographic data including work schedule, as well as the clinical course and treatment history for MDD. Among these features three deserve emphasis.

The major contributor to MDD risk is environmental, and without information about the environment it is hard to see how we can interpret genetic signals. A key unanswered question in MDD genetics is how best to obtain information about the relevant environment. Second, depression is a recurrent illness: up to 85% of cases in specialized mental health care and in primary care will experience recurrence; in the general population the rate is lower, but still high: up to 35% [131]. Despite its importance, understanding the factors that contribute to recurrence is an area yet to receive the attention of geneticists. Finally, the lack of deep symptomatic profiles is the most egregious omission in genetic studies of MDD. Central to MDD are episodic severe shifts of mood, together with neurovegetative and cognitive changes [43, 132, 133]. We need to document these unique features of MDD and to identify which loci contribute to their risk.

If it is a hard task to obtain thousands of interview-based, diagnoses, then it would appear even harder to collect the additional information. We can however improve the current data sets by taking advantage of the information accumulating in Biobanks. Many phenotypes in biobanks correlate with MDD, and these can be used as proxies for information we are missing. As a demonstration of this we analyzed the UK Biobank, taking the CIDI-SF based Lifetime MDD phenotype to represent a gold-standard assessment [126]. We then imputed Lifetime MDD in the entire cohort, using 216 other phenotypes in the biobank, chosen regardless of their putative relationship with MDD, using SoftImpute [134] (a variant of principal component analysis that accommodates missing data, and uses observed phenotype data to identify latent factors). We were able to show that the top phenome-wide factors capture pleiotropic axes for MDD, allowing us to identify genetic effects that are specific to lifetime MDD (which stood in for the gold standard MDD cases) [126]. Remarkably, we found that the one-item self-assessment measures (which capture general dysphoria), residualized of these latent factors, index core, MDD-specific biology. In short, we can dissect MDD into two components: shared pleiotropic factors and core factors. Both classes of derived phenotype are heritable, with the former defining a highly polygenic background of mental health and social factors, and the latter defining a less polygenic signature of core MDD biology. However, currently our imputation methods do not supply rich phenotypic data about specific symptom patterns, or features of the course of illness.

In discussing how to improve depression measurement, Fried pointed out “we cannot divorce our measures of depression from our theories about what depression is” [120]. It’s notable how few theories we have about the nature of depression. In part this might be because attempts to replace DSM criteria with neurobiological constructs (the NIH Research Domain Criteria RDoC) [135] have not progressed well. Despite the collection of relevant behavioral, genetic, and neuroimaging data, achieving transformative progress proved more difficult than expected [136]. In part it reflects the complexity of depression. In a review of risk factors Kendler identified 37 potential causes [45] (as he points out, not much less than the 44 identified by Richard Burton in 1621 [137]).

There are sources for new theories about the nature of depression, but these so far have not been exploited in genetic research. One comes from advances in neuroscience, that enable us to explore cellular and molecular mechanisms by deploying genetically encoded reagents and imaging technologies in animals. For example, investigation of how ketamine has its effect has shown that it reduces bursting in the lateral habenula, an effect isolated to one cell type (astrocytes) and indeed one channel in that cell type: a potassium channel, Kcnj10, that provides a molecular clue to the etiology of at least one form of MDD [138, 139]. Human genetic studies have yet to determine whether risk loci act through this mechanism. Such a discovery could be transformative.

A second source of new theories of depression comes from psychology. Moving away from a somewhat stale debate about the values of categorial versus dimensional categorization, Borsboom proposed a network theoretical description of depression [140, 141], arguing that the probability of a change from a normal to a depressed state is related to elevated temporal autocorrelation, variance, and correlation between emotions in fluctuations of autorecorded emotions [142]. Translating these concepts into genetically testable ideas is an important challenge to the field.

The diverse symptomatology, the way MDD is seen to arise from different starting environmental points, from childhood trauma through to adult-onset adversity, its comorbidity with many different chronic diseases, together with hints of multiple, diverse biological causal pathways, all support an etiological heterogeneity that is at odds with claims that its genetic basis is primarily pleiotropic and held in common with many other diseases. There are ways forward, as I have outlined, similar to those that propelled success in cancer research [143]. Understanding the origins of cancer progressed from careful clinical observation, for example by noticing the effects of folate deficiency on blood cells. For MDD we need new cohorts, more complex, deeper phenotypes, combined with the use of existing data sets, but most crucially we need ideas about the nature of the condition, so that we ask and answer the right clinical questions: what are the different forms of the disorder? What are the characteristics of each? And how can we best treat each form as we discover it and its causes?
